# Temporal-Microclimatic Factors Affect the Phenology of *Lipoptena fortisetosa* in Central European Forests

**DOI:** 10.3390/ani10112012

**Published:** 2020-11-01

**Authors:** Remigiusz Gałęcki, Jerzy Jaroszewski, Xuenan Xuan, Tadeusz Bakuła

**Affiliations:** 1 Department of Veterinary Prevention and Feed Hygiene, Faculty of Veterinary Medicine, University of Warmia and Mazury in Olsztyn, 10-719 Olsztyn, Poland; bakta@uwm.edu.pl; 2 Department of Pharmacology and Toxicology, Faculty of Veterinary Medicine, University of Warmia and Mazury in Olsztyn, 10-719 Olsztyn, Poland; jerzyj@uwm.edu.pl; 3 National Research Center for Protozoan Diseases, Obihiro University of Agriculture and Veterinary Medicine, Obihiro 080‐8555, Japan; gen@obihiro.ac.jp

**Keywords:** deer ked, ecophysiology, ectoparasite, louse flies, temporal-climatic variation, vector, phenology

## Abstract

**Simple Summary:**

The population of *Lipoptena* spp. continues to increase in Central Europe. Deer keds are obligatory hematophagous ectoparasites. Environmental conditions play a large role in the prevalence of these insects. However, the relationship between environmental conditions and population size has not been assessed in *Lipoptena fortisetosa*. The objective of this study was to find a link between *L. fortisetosa* flights and selected weather conditions in forests. Insects were sampled, and selected climatic factors were measured. The correlations between the number of insects and the examined factors were calculated with the use of statistical methods. The results suggest that the abundance of ectoparasites is correlated with time, temperature, relative humidity and wind speed. The beginning of ked flights could also be associated with variations in climatic conditions. In the future, these relationships can be used to minimize the negative impact of keds on humans, livestock and companion animals.

**Abstract:**

The objective of this study was to determine the correlations between the abundance of *Lipoptena fortisetosa* on new potential hosts and selected temporal-microclimatic conditions in a forest at the beginning of the host-seeking period. Louse flies were collected between 6 May and 15 July of 2019 and 2020 in a natural mixed forest in Poland. Keds were collected by three investigators walking along the same forest route during each sampling session. The number of captured keds and the date (time), temperature (°C), relative humidity (%), air pressure (hPa) and wind speed (km/h) were recorded. A total of five measurements were performed during each sampling session. The influence of temporal-microclimatic conditions on the number of collected ectoparasites was evaluated with the use of a Generalized Additive Model (GAM). A total of 1995 individuals were obtained during field surveys. The results of the GAM revealed a correlation between the number of host seeking *L. fortisetosa* vs. time, temperature, relative humidity, and wind speed. An increase in temperature was most highly correlated with the abundance of louse flies in the environment.

## 1. Introduction

The presently observed climatic variations have led to changes in animal population dynamics as well as the appearance of new species in locations where they have never been known to occur [1,2,3]. These processes have significantly increased the population of ectoparasites in Europe. The population of flies of the family Hippoboscidae has also increased over the years. New ked migration patterns have been observed, for example, the migration of louse flies to Fennoscandia. Keds have been migrating to Fennoscandia from the southeast since the early 1960s. The northern distribution limit currently lies at approximately 65° N and is gradually spreading northwards [4,5]. 

Keds are obligatory hematophagous ectoparasites of birds and mammals. They belong to the family Hippoboscidae, which comprises 21 genera, of which 4 feed primarily on mammals (including *Hippobosca* spp. and *Lipoptena* spp.) and 17 feed on birds. The genus *Lipoptena* spp. consists of 32 species. The species that are of greatest concern in veterinary medicine include *L. cervi*, *L. capreoli* and *L. fortisetosa,* which are commonly encountered in Europe, Siberia, China and North America, as well as *L. depressa*, *L. mazamae* and *Neolipoptena ferrisi,* which can be found in North America. Flies of the genera *Lipoptena* and *Neolipoptena* shed their wings after finding the definitive host. Keds are highly host-specific, and they breed only on wild ruminants. Louse flies are univoltine insects that reproduce by adenotrophic viviparity. Females produce larvae that do not have mouth hooks. The larvae molt several times; the last molt is retained, and it hardens on the pupa to form a puparium. Pupation lasts around 1 h, and pupae remain on the host’s body or fall to the ground. The life cycle of keds is probably determined by climate conditions, and it lasts up to 30 days in a warm climate and 270–370 days in a temperate climate with winter diapause [6,7]. Soon after emergence, adult flies need to find a host in order to survive and breed.

The prevalence and intensity of *Lipoptena* spp. infestation in specific hosts (cervids) can be very high. In Poland, large numbers of cervids and other animal species have been found to be infected with this ectoparasite. Deer keds were identified in 64% of European roe deer [8], 76% of fallow deer and 78% of European red deer [9]. Louse fly infestations have also been reported in the European bison [10] and in nonspecific hosts such as companion animals [2]. These ectoparasites also colonize livestock [11]. *Lipoptena* spp. can land on and bite humans [12,13]. Humans bitten by these ectoparasites are at risk of dermatitis, allergic rhinoconjunctivitis or even anaphylactic shock. Deer keds pose a potential threat of horizontal transmission of pathogens [14,15,16,17].

Environmental conditions play a large role in the abundance of *Lipoptena* spp. It should be noted that weather conditions can also significantly affect the behavior and physiology of keds [18]. *Lipoptena cervi* pupae are able to survive winter even in the cold regions of northern European [19]. Research indicates that these ectoparasites have developed strong adaptive mechanisms to low temperatures. Härkönen et al. [20] reported that *L. cervi* can survive in adverse conditions even without an acclimatization period. Keds caught in late autumn were characterized by a significantly higher ratio of unsaturated to saturated fatty acids, which probably enhances the fluidity of lipid membranes and promotes the maintenance of protein functions at low ambient [21]. Due to higher concentrations of free amino acids after cold acclimation, keds could be a freeze-tolerant species with a supercooling point of −7.8 °C and a lower lethal temperature of −16 °C [22]. The above not only contributes to ectoparasite migration to colder areas, but it can also induce changes in their seasonal abundance and increase ked survival rates due to higher tolerance to low temperature. However, these factors have never been investigated in *L. fortisetosa*.

Previous studies confirmed the role of temperature and annual variations in climate on the abundance of *L. cervi* [19,23]. However, most research was based on data from weather stations and meteorological centers. Meanwhile, the microclimate of specific ecosystems such as forests may differ from meteorological data [24,25]. Differences in microclimate can play an important role even in small areas and forests on account of their specific topography [26,27]. The forest is the natural biotope for *L. fortisetosa,* but the factors that influence the abundance of keds in forests have never been identified. There are no reports describing the impact of climatic conditions at the beginning of the flight activity season on the prevalence of *L. fortisetosa.* Differences in gender distribution over time have never been investigated. Moreover, the impact of climate on *L. fortisetosa* has never been analyzed in Central Europe.

The objective of this study was to identify the correlations between the abundance of *L. fortisetosa* among new potential hosts and selected temporal-microclimatic conditions in forests at the beginning of the host-seeking period.

## 2. Materials and Methods

### 2.1. Sample Collection

Louse flies were collected between 6 May and 15 July of 2019 and 2020 in a natural mixed forest in Olsztynek municipality, Warmian-Masurian voivodeship in Poland (53°40′31.4″ N 20°20′00.4″ E). This study period was chosen because it did not coincide with the animal breeding season or forest management activities and it was characterized by a noticeable increase in entomofauna activity. Coniferous trees, mainly Scots pine, were predominant in the studied forest. The underwood and undergrowth consisted of plant species typical of the local climate and geographic zone. The undergrowth was covered with a layer of herbaceous plants and moss. The research area was a habitat of a large cervid population, including red deer, European roe deer and moose. The forest also met all habitat characteristics and did not feature any negative factors associated with the intensity of ked activity, described by Madslien et al. [28]. Keds were collected by three investigators walking along the same forest route during each sampling session. The exact route had been determined before the experiment, and it spanned a distance of 5.81 km based on GPSMAP^®^ 62 readouts (Garmin, Olathe, KS, USA). The altitude range was between 151 and 158 m above sea level. The sampling route intersected animal migration routes, and fresh traces of animal activity were found. Human activity that could potentially influence the results was not observed. A total of 140 observations were carried out. The measurements were performed between 1:00 p.m. and 5:00 p.m. (data were not recorded outside this time interval). The investigators walked for 15 minutes and then paused for 10 minutes. They wore brown cotton clothing covering the entire body and collected keds immediately after they had landed on the clothing. Similar methods are used to sample ticks and ectoparasites [23,29]. The acquired ectoparasites were placed in separate test tubes.

### 2.2. Species Identification

Ked samples were transported to the Biological Hazard Laboratory at the Faculty of Veterinary Medicine of the University of Warmia and Mazury in Olsztyn. Ked species and sex were identified based on their morphological characteristics under the Leica M165C stereoscopic microscope. Louse flies were identified to species level based on body dimensions, wing venation, length and structure of palpi, and the number of erect hairs on the mesonotum [6,30,31]. Samples containing species other than *L. fortisetosa* were eliminated from further analysis.

### 2.3. Microclimate Measurements

The number of collected keds, and the sampling date (time in days), temperature (°C), relative humidity (%), air pressure (hPa), and wind speed (km/h), were recorded. The measurements were performed before each sampling session, every hour during the field trial, and after its completion. A total of five measurements were performed during each sampling session. Selected microclimate parameters were measured with the ST−8820 Multi-Function Environment Meter (Cem, Shenzhen, China) (temperature, relative humidity), Testo 511 manometer (Testo SE and Co. KGaA, Lenzkirch, Germany) (air pressure) and Kestrel 2000 anemometer (Kestrel Instruments, Boothwyn, PA, USA) (wind speed). The devices had been calibrated by the manufacturers before the experiment. All measurements were performed at shoulder height by the same investigator.

### 2.4. Statistical Analysis

The assumption of linearity and normality was checked before the statistical analysis. Two-dimensional scatter plots of the analyzed variables were generated to examine linearity. The assumption of normality was validated with the use of histograms and normality charts for residues. Statistical anomalies were ruled out with the k-nearest neighbors algorithm. Statically significant differences between the sex distribution of *L. fortisetosa* and time were determined with the use of the General Linear Model (GLM). Linear correlation models (Pearson’s correlation coefficient—*r*) were built to illustrate the correlations between temporal-microclimatic conditions and the number of collected *L. fortisetosa*. The calculated values of *r* were interpreted as follows: below 0.2—absence of correlation, 0.2–0.4—weak correlation, 0.4–0.6—moderate correlation, 0.6–0.8—strong correlation, 0.8–0.9—very strong correlation and 0.9–1.0—nearly perfect correlation. The influence of temporal-microclimatic conditions on the number of collected ectoparasites was evaluated with the use of the Generalized Additive Model (GAM). The model was built to maximize the prediction quality of the dependent variable (abundance of deer keds) based on the analyzed covariates (time, temperature, relative humidity, air pressure and wind speed). The following statistical descriptors were calculated for the collected samples: mean (*M*), median (*ME*), standard deviation (*SD*), standard error (*SE*), variance (*V*), minimum (*Min*), maximum (*Max*), kurtosis (*K*) and skewness (*Ske*). Differences were regarded as significant at *p* < 0.05. Data were processed in the Statistica 13.3 program with a medical application (TIBCO Software, Palo Alto, CA, USA).

### 2.5. Model Validation

The developed Generalized Additive Model was evaluated (without the time covariate) in four different locations (I—53°39′34.2″ N 20°30′46.0″ E; II—52°09′59.9″ N 18°18′55.9″ E; III—52°11′27.6″ N 17°51′53.0″ E; IV—53°28′24.8″ N 20°34′04.9″ E). In each location, three measurements were made according to the sampling and measuring protocols described above.

## 3. Results

**Figure 1:**
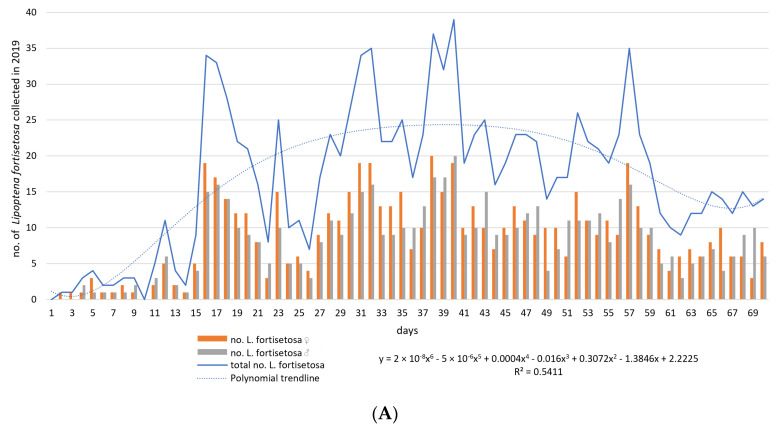

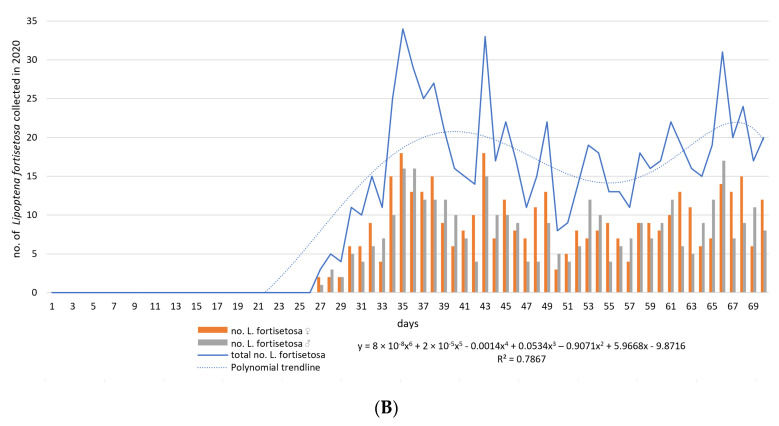
The number of *Lipoptena fortisetosa* specimens collected on each sampling day between 6 May and 15 July in 2019 (**A**) and 2020 (**B**). Legend: y—logarithmic function; R^2^—coefficient of determination.

The first louse flies to be identified as *L. fortisetosa* were collected on 7 May 2019 and 2 June 2020. During field tests, a total of 1995 individuals (*M* = 14.25; *ME* = 14.5; *SD* = 10.96; *SE* = 0.93; *V* = 76.87; *Min* = 0; *Max* = 39.0; *K* = −0.94; *Ske* = 0.23) were sampled, including 1028 (51.53%) females and 967 (48.47%) males. Differences in sex distribution as a function of time were not statistically significant (for the model: *df* = 1; *MS* = 225.04; *F* = 5.38; *p*-value = 0.02; female vs. male: *df* = 1; *MS*= 2.61; *F* = 0.78; *p*-value = 0.38; male vs. female vs. time: *df* = 1; *MS* = 0.047; *F* = 0.014; *p*-value = 0.91). The first peaks denoting high levels of ked activity were observed on day 16 in 2019 and day 35 in 2020. The number of louse flies collected on each sampling day is presented in Figure 1.

A very high linear correlation was noted between temperature and the abundance of *L. fortisetosa*. Relative humidity was bound by a high linear correlation with ked flights. A moderate linear correlation was determined between time and the prevalence of *L. fortisetosa*. A negative moderate linear correlation was observed between wind speed and louse abundance. No significant correlations were noted between *L. fortisetosa* abundance and air pressure. The results of the correlation analysis are presented in Table 1.

**Figure 2:**
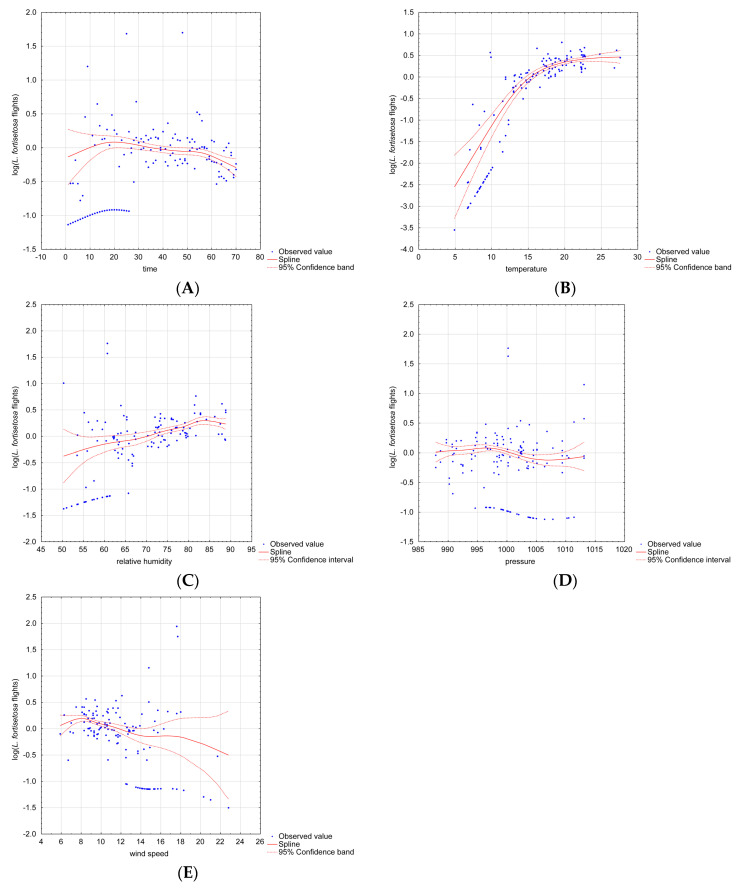
Nonlinear effects of different continuous covariates on *Lipoptena fortisetosa* abundance. Legend: the plots present the results of two-dimensional cubic spline interpolation with the observed predictor values plotted against the partial residuals; (**A**) time, (**B**) temperature, (**C**) relative humidity, (**D**) air pressure* and (**E**) wind speed; *—not significant.

The results of the GAM indicate that time, temperature, relative humidity and wind speed significantly influenced ked flights. The possible nonlinear effects of continuous covariates when the abundance of *L. fortisetosa* was taken into account are presented in Figure 2. The prevalence of *L. fortisetosa* increased significantly between days 15 and 20 (Figure 2A), and a minor decrease was observed on the following days. Temperatures below 5 °C had a negative effect on *L. fortisetosa* abundance. The conducted measurements indicate that keds thrived at a temperature of 17–22 °C (Figure 2B). The GAM revealed that above-optimal temperatures may not exert a positive effect on louse prevalence. In the model, ked abundance was also somewhat correlated with relative humidity. The number of recorded ectoparasites tended to increase with a rise in humidity. Ked abundance was relatively stable within the humidity range of 70–80% (Figure 2C). The optimal wind speed for *L. fortisetosa* was 4–12 km/h (Figure 2E), and higher wind speeds exerted a negative effect on ked abundance. Air pressure was not significantly correlated with the prevalence of *L. fortisetosa* (Figure 2D). The results of the GAM are presented in Table 2. The results of model validation are presented in Table 3.

## 4. Discussion

The population of *L. fortisetosa* continues to increase in the natural environment. These ectoparasites are also found in nonspecific environments such as cities [2]. In the future, the size of *L. fortisetosa* populations is likely to increase, and the species could also be expected to colonize new ecosystems, due to both climate change and the penetration of wild ruminants into rural/city areas. The phenology of the analyzed ectoparasites is one of the most important considerations in epidemiological monitoring. The phenology of *L. cervi* has been described in detail in Northern Europe [29]. However, no such research has been conducted in Central Europe (research site), where the population of *L. fortisetosa* continues to grow. Our previous study conducted in 2017–2018 confirmed the occurrence of *L. fortisetosa* in May, but accurate microclimatic measurements were not performed. These findings differ from the observations made in other species of louse flies. The variations in ked phenology could be linked to an increase in temperatures in Europe [32,33]. Monitoring programs in Central Europe have revealed an increase in average temperatures, in particular between January and August, as well as temperature extremes between 1961 and 2010 [34].

This study confirmed the presence of correlations between temporal-microclimatic factors and number of flies seeking new hosts. A strong relationship was noted between temperature and the abundance of flies. The time of measurement was also a significant factor, but it was far less correlated with the number of collected flies than temperature. A similar relationship was noted between the abundance of *L. fortisetosa* and humidity. A minor decrease in the size of the collected samples was observed on successive days of field tests, which points to the absence of new generations. Sex distribution was not correlated with the time of measurements. 

Similar correlations between temperature and abundance of *L. cervi* have been described by Härkönen et al. [19], Mysterlund et al. [23] and Välimäki et al. [35]. The cited studies were conducted in regions with a different climate and different animal populations than in Poland. Temperature could significantly affect the developmental cycle of *L. fortisetosa* by disrupting winter diapause. Multiple pathways are associated with diapause completion, and the stages of the developmental cycle are modified by environmental conditions [36]. Hodek and Hodková [36] reported that diapause is successfully completed at intermediate or high temperatures, and can even be stimulated by high temperature. The beginning of the flight activity season can also be influenced by cryoprotectants [21,22]. In the future, the continued emergence of cryoprotectants could alter the correlations between the abundance of *Lipoptena* spp. and environmental factors. Cryoprotectants can also contribute to a rise in the population of *L. fortisetsa* by decreasing the mortality of pupae in winter. However, cold temperature plays a certain role in the life cycle of deer keds. Härkönen and Kaitala [37] argued that *L. cervi* pupae require longer exposure to high temperatures to terminate diapause if the cold period is short. This phenomenon could be the main reason behind the observed differences in phenology. High temperatures most probably prolonged winter diapause.

Photoperiodism could be responsible for the correlation between time and *L. fortisetosa* flights. However, the emergence of adult *L. cervi* was not correlated with two photoperiods under laboratory conditions [38]. In contrast, Mysterud et al. [23] speculated that photoperiod could be an important factor under field conditions. Light exposure plays an important role in other Hippoboscidae ectoparasites. For example, *Pseudolynchia canariensis* emerge only in selected photoperiods [39], whereas *Hippobosca equina* flights are more frequent at certain times of the day [40]. The importance of photoperiod could also be associated with the strong correlation between measurement date and temperature. Further research is needed to investigate the effect of photoperiod on *L. fortisetosa* activity.

The results of the present study also suggest that ectoparasite activity could be correlated with variations in relative humidity. Moyer et al. [41] reported that abiotic factors such as relative humidity can induce significant changes in ectoparasite pressure on the host population. Low humidity can inhibit pupal development [38]. According to Bakhtushkina [42], the lifespan of *L. cervi* increases with a rise in humidity. Ectoparasites have the longest lifespan within a humidity range of 60–80% and a temperature range of 14–16 °C. Under laboratory conditions, pupal development lasts 90 days on average at a temperature of around 20–25 °C and estimated humidity of 60–80% [42]. Our research suggests that similar relationships could occur in a forest environment. The optimal humidity and temperature of ambient air for the survival of *L. cervi* imagines were established at ≥70% and 2–5 °C, respectively, by Popov [43]. Therefore, relative humidity could be associated with the number of *L. fortisetosa* seeking new hosts.

A significant correlation between air pressure and ked abundance was not observed in this study. The impact of air pressure on insect activity has been confirmed by other authors. According to Wellington [44], a decrease in barometric pressure stimulates general activity in muscoids. However, the sensitivity of the receptors responsible for monitoring air pressure may vary between insect species [45]. McFarlane et al. [46] found that *Frankliniella schultzei* was capable of detecting changes in atmospheric pressure. In insects, the mechanisms for monitoring air pressure could have evolved to offer protection against adverse weather events. Despite the absence of significant correlations between air pressure and insect abundance, this mechanism cannot be ruled out in the current study. However, further research is needed to investigate the responses of *L. fortisetosa* within a wider range of air pressure values. 

The calculated values of *r* and the results of the GAM revealed a significant correlation between the number of host-seeking *L. fortisetosa* and wind speed. According to Härkönen et al. [19], the suitability of northern habitats for ambushing hosts, especially in the treeless tundra, could be compromised by difficult flying conditions, such as strong winds. Research carried out in eastern Finland by Paakkonen et al. [47] confirmed that the number of deer keds is inversely correlated with windiness. The presence of such a relationship can also be inferred from the behavior of reindeers, which use windy mountaintops primarily to avoid ectoparasite harassment [48].

The GAM was applied in the study to account for the relationships between the analyzed covariates. However, the presented correlations do not necessarily prove a direct cause-and-effect relationship. It is possible that the described factors are correlated with an unknown variable or a set of variables. The number of sampled keds as well as the beginning of the flight activity season could be influenced by more profound changes in a system of complex environmental (e.g., deer migrations) and weather (e.g., insolation) variables.

## 5. Conclusions

A thorough knowledge about the activity of *L. fortisetosa* is needed to address the steady increase in the population of these ectoparasites and seasonal changes in their abundance. Climate change could be an important factor in the observed phenomena. The variations in selected microclimatic factors were correlated with the number of host-seeking keds in the environment. Higher temperatures were strongly correlated with an increase in ked abundance in the surveyed forest. In the group of the studied factors, temperature was primarily responsible for the beginning of the flight activity season of *L. fortisetosa*. However, the presented correlations are not necessarily indicative of a cause-and-effect relationship. These ectoparasites are potential pathogen vectors, which is why their population, behavior and phenology should be regularly monitored. Further research should focus not only on the epidemiology and spread of *L. fortisetosa*, but also on the effectiveness of repellents. Environmental observations should be carried out to determine the number of new generations in one flight activity season in a changing climate. In the future, information about the phenology of the species and the relationships between the described factors can be used to develop preventive programs and control ked infestation in the environment. Preventive measures should also be implemented to minimize the negative impact of keds on humans, livestock and companion animals.

## Figures and Tables

**Table 1:** Correlations between temporal-microclimatic conditions and the abundance of *Lipoptena fortisetosa* (Pearson’s correlation coefficient *r*).

Correlations Between the Studied Factors		Values	
		*p*-Value	*r*
Number of sampled *Lipoptena fortisetosa*	time (days)	<0.001	0.45
	temperature (°C)	<0.001	0.84
	relative humidity (%)	<0.001	0.76
	air pressure (hPa)	0.15	−0.19
	wind speed (km/h)	<0.001	−0.59

Legend: *p*-values < 0.05 were considered significant.

**Table 2:** Parameter estimates in the Generalized Additive Model (GAM) analyzing the correlations between temporal-microclimatic factors and *Lipoptena fortisetosa* abundance.

Parameters *n* = 140	Values				
	*df*	GAM coef.	*SE*	*SS*	*p*-Value
Intercept	1	1.12	0.23	4.82	0.005
Time	4.15	−0.0054	0.0013	−4.21	0.038
Temperature	4.06	0.057	0.0068	8.39	<0.001
Relative humidity	4.02	0.017	0.0029	5.29	0.041
Air pressure	4.04	−0.0086	0.0035	−2.42	0.074
Wind speed	3.94	−0.026	0.0088	−3.04	0.003

Legend: *n*—number of measurements; *df*—degrees of freedom; GAM coef.—Generalized Additive Model coefficient; *SE*—Standard Error; *SS*—Standard Score; *p*-values < 0.05 were considered significant.

**Table 3:** Validation of the GAM in different locations.

Location *n* = 12	Temperature		Relative humidity		Air pressure		Wind speed	
	°C	log(*L. fortisetosa* flights)	%	log(*L. fortisetosa* flights)	hPa	log(*L. fortisetosa* flights)	km/h	log(*L. fortisetosa* flights)
I	18.1	0.29	72.6	0.11	1002.4	0.44	13.3	−0.17
	19.5	0.40	77.8	0.17	1001.2	0.48	14.7	−0.26
	15.2	−0.06	68.2	0.07	998.5	0.22	8.2	0.28
II	19.3	0.27	62.4	−0.06	1001.6	−0.19	15.5	−0.07
	17.9	0.23	64.3	−0.13	997.3	0.05	8.0	0.34
	20.6	0.36	67.7	0.002	1000.6	0.12	10.2	0.09
III	22.3	0.41	74.8	0.29	1002.7	−0.29	8.9	0.05
	21.7	0.34	76.0	0.14	1005.2	0.33	6.8	0.21
	18.8	0.12	71.9	−0.03	1003.7	−0.16	10.6	0.04
IV	22.3	0.33	67.8	0.05	1000.8	−0.31	16.1	−0.32
	17.4	0.12	70.4	−0.08	999.2	0.25	10.3	0.08
	19.1	0.28	71.3	0.16	−0.07	1003.4	0.05	10.2

Legend: *n*—number of measurements; I-IV—surveyed locations.
